# Transactions of the American Medical Association

**Published:** 1855-01

**Authors:** 


ART. IV.— *Transactions of the American Medical Association. Report of the Committee on Medical Education.*

This Report is from the pen of Dr. J. L. Cabell, of Virginia, the chairman of the committee.

Dr. Cabell evidently belongs to the reform party in our educational system, and believes that numerous and radical changes are necessary in order to secure an adequate standard of acquirement in candidates for the doctorate.

He assumes in the first place, what he does not sufficiently prove, that the profession, by reason of its insufficient attainment, has lost in the dignity of its social position, and no longer receives an homage which it formerly obtained.

Perhaps this "homage" is somewhat hypothetical. If the physicians of the olden time were the objects of so great respect, it was not as physicians, but as men of varied learning and studious habits. So far as medical attainment is concerned, we hold with Macauley, that the bricklayer, falling from a scaffold to-day, is the recipient of a higher order of surgical and medical skill than Queen Elizabeth could have procured for her own ailments in her day. The estimation of the profession at the present day rests, then, not upon its scholarship in general, but upon its ability to relieve the ills falling to its care.

Again, the prestige of *professional* name is now no more. It has been set aside for other and more important considerations. To be an authorized attorney, a legitimate clergyman, or a veritable M. D., helps no man farther than to make him a *candidate* for the good-will of the public. This somewhat capricious body may refuse to consider the claims of a man until he presents his diploma; but, no matter how conspicuously he may display the valuable parchment, it still looks at the man rather than the sheepskin, at the individual rather than the class, and according to the estimate it forms of him, and him only, is it governed in the "homage" it may render.

Dr. Cabell, in his first resolution, reaffirms the previous action of the association relative to prolonged terms of lectures, preliminary education, and the elevation of the standard for gradation.

The prolonged term of lectures has been a favorite theory of the reformists. The arguments in its favor are certainly solid. A large share of the profession will think with Dr. Cabell that the collegiate course should be the essential part of medical teaching, and that private preceptorship should assume a minor importance.

The difficulty here is in a want of concert among the schools. At first thought it would appear that those schools which are deriving heavy incomes from their pupils should be the first to adopt the lengthened term, as they could best afford it. But it happens that no such disposition is manifested by them. They are content with their present position. If the income of a school is $30,000 for a four months' course, it would be folly to work six months for the same money. This is the real position of the leading schools. They are fully aware that they cannot increase their fees, or extend their term, without a loss of students and money, and however earnestly the reform party may urge their really sound arguments, they are whistling down the wind. Of course the smaller schools must follow their lead. *They* are not able to make the sacrifice, or, if in a spirit of zeal they once do so, they are compelled, by the strong argument of money, to return to their original position.

As to preliminary education all seem to be agreed upon its importance. It should undoubtedly be made a requisite, but who shall make the necessary examination? We are told that it must be made before the entrance of the student in the office of his preceptor. But when preceptors neglect their duty, as happens in nine cases out of ten, who shall shut him out of the profession?

This pleasant duty is assigned to the colleges, and here rests the whole question. It is agreed on all hands that the colleges shall be the scape-goats — all the sins of the entire profession are to be laid upon them.

Now we assume that the college has no right or duty to go behind the certificate of the preceptor. Colleges are usually very good-natured institutions, but to insist that they shall shoulder the whole responsibility of making good doctors of all the students — good, bad, and indifferent — is a little too much for their long-suffering tempers. We know some capital teachers, men of the highest medical skill combined with great success in teaching, who would stumble very many times before they reached the gerund in conjugating the verb *amo*.

It is a question yet to be considered whether the duty of the college extends any farther than the rejection of such candidates as may pass an unsatisfactory or insufficient examination. The protection of the character of the profession is a duty which rests upon itself, and cannot be delegated to the colleges.

The standard of attainment necessary to graduation, is a matter which it is somewhat difficult to regulate by any legislation. Two things would seem to be necessary to this end, viz: 1st. A conscientious and intelligent discharge of their duties by the examining professors; and 2d. Some measure of the mind of the student, by which the colleges may be able to judge between his apparent and real attainments; some guage, which shall inform them whether the studious habits of the tyro are to be continued into professional life; in other words, whether he has been reading for a diploma, or for usefulness in after life. A year or two since, a well-known teacher, after examining a candidate, declared that he *must* pass him, for he had answered all questions correctly, but he was at the same time conscious that the man was entirely unfit for the active duties of practice. There is no help for such cases.

This is practically acknowledged by Dr. Cabell in his recommendation of a board for final examinations, who shall pass upon the merits of the student *after* he has received his diploma. This, while it would considerably depreciate the value of a college diploma, would, perhaps, to some extent, remedy the evil. Heretofore our colleges have been entrusted entirely with this matter. The possession of a diploma entitles its possessor to admission to the county societies without any examination. The appointment of an examining board by the state or county societies would, probably, have the effect of making colleges more careful.

But here arises the old difficulty. A state board of examiners would find their whole time absorbed if they examined all the graduates, and it would be necessary to make a salaried office for them to discharge their duties properly. Who is to pay the salary? How are students already in possession of a diploma to be persuaded to go voluntarily before a distant and dreaded board of censors, when they have already all the privileges of an M. D.?

We confess that we have only shown the difficulties which environ this subject, without pretending to remedy the real and acknowledged evils which attend the present system. But it is a question of grave importance, whether these evils belong to the system itself, or whether they are not simply defects in its practical working, to be remedied by a more careful and conscientious performance of their duties by the colleges.

So far as we have any knowledge of examinations for degrees, we have witnessed a sufficient number of rejections to convince us that the graduation fee is not the chief consideration. We recollect an instance where one-fourth of the candidates were rejected.

What, then, are the duties of the colleges? To teach as thoroughly and practically as possible; to furnish means for practical and clinical instruction; and, finally, to graduate only those who in their opinion are fitted for the duties of the physician. So far as terms of study are concerned, they are not necessarily important. It is fair to assume that three years of study and two courses of lectures are necessary to a fair degree of attainment. Some men may, by great effort, come up to the standard at the close of a single term, but another will benefit them, and no exceptions should be made to the rule.

In the recommendation of "private schools," every thinking mind must concur; but it is nevertheless extremely difficult to sustain them. Attached to a chartered college they may be made profitable as a nucleus for winter classes, but the history of private effort, even in our largest cities, shows that it cannot yet be sustained without some such motive.

Dr. Cabell also insists upon the establishment of separate chairs of physiology and medical jurisprudence in the schools. How many of the schools are wanting such chairs we do not know, but it is very evident that physiology should be thoroughly and experimentally taught.

We are glad to notice that Dr. Cabell differs somewhat from Dr. Pitcher in his estimate of the value of clinical instruction. In order to enhance the merits of his favorite school, Dr. Pitcher, probably unconsciously, but still unfairly, depreciated the value of clinical instruction. He had a fair field for attack in the fuss and parade made in "our great medical centre," over a hospital two miles off and closed to students, but this was no fair argument against the system itself.

While we have been penning this critique, a part of the graduating class of the Buffalo Medical College have been employed in diagnosticating the position of the *fœtus in utero* by the sounds of the fœtal heart, and each of them will, during the winter, have the opportunity of attending an accouchment under the guidance of his teacher.

All have cases of disease assigned to them, of which they are required to furnish to the professor of practice a written history, diagnosis, and treatment, for his inspection. It is only by such means that clinical teaching can be made truly useful. Commenced, as it has been in the Buffalo school, early in the term, and continued to the end, each student will graduate with some experience already in store, and its value cannot well be over-estimated.

In conclusion we regard Dr. Cabell's report as much more practical, and much less biased by local prejudices, than its predecessor. At the same time we can trace the Virginian tendency to abstractions, and while we are compelled to regard a portion of Dr. Cabell's propositions as utterly impracticable, we are glad to recognize the earnest spirit of professional pride and zeal which has dictated every line of this able and scholarly report.

*Report of the Committee on the Epidemics of Kentucky and Tennessee.* (By Drs. W. L. Sutton, E. B. Haskins, T. Lipscomb, and F. A. Ramsey.)

Dr. Sutton is probably the principal writer of this report. It is always difficult to give to such an essay that degree of merit which the importance of its subject demands. The want of proper registration laws leaves to amateurs the whole labor of collecting medical statistics, and subjects them to all those sources of error to which imperfectly-collected facts are liable. Those at all familiar with statistical research will at once recognize this difficulty.

The history of these epidemics is collected by individual effort. Of course only here and there a township in these two great states is represented, and in them, only the cases of a single physician are usually reported. Time, labor, and expense, the three great necessities of statistical research, are all wanting in this system.

Notwithstanding these obstacles, the committee have given us a report which possesses a high value, and is, moreover, extremely interesting. Wherever accurate data have been attainable they have been presented.

In the record of the mortality of Memphis, the committee call attention to the fact that phthisis and pneumonia make up almost one-fourth of the fatal diseases of that southern city. This is in accordance with the expressed opinion of quite a number of medical observers, even at points much farther south than Memphis. It would seem that climate has not that degree of influence in its causation which has been assigned to it.

Heretofore Charleston, S. C., has been considered a favorable latitude for consumptives, and large numbers of invalids have yearly flocked to places no farther south for a fancied relief. The history of many of these cases is truly mournful. Leaving behind them the comforts of a northern home, and the solicitous care of friends, they wander off to a land of strangers, and subject themselves to hardships peculiar to a southern winter, and inconveniences peculiar to southern habits, until, at the coming of the spring with its exhausting heats, they find a grave in a southern soil.

Were these facts fully appreciated, did medical men at the north fully explain to their patients that consumption and pneumonia are scarcely less frequent at the south than at the north, we should have less of this breaking up of home associations in a vain search after health. There is no good reason why there should be any more phthisis at the north than at the south. It is not in any correct sense a disease of climate, and is not to be cured by change of air merely.

The year 1853 was not attended by any severe epidemics in Kentucky and Tennessee. The unusual health of the river towns was ascribed to the fact that the "June rise" of the Mississippi did not occur as usual. The conclusions of the committee are, from all the facts within their reach, that malarial influences exert an active part in the causation of summer and autumnal diseases of the south.

The meteorological conditions receive some consideration, but unfortunately the observations made are neither full nor accurate. Enough is ascertained, however, to render it probable that the humidity of the air has a considerable share in the production of zymotic disease, but as none of the observers have given the dew-point range, or the amount of rain which has fallen, we are unable to draw any reliable positive inferences.

A negative inference may be obtained from the records of temperature. The average number of deaths in the city of Memphis, monthly, was 34; the total of deaths for the year being 410. Of these deaths 51 occurred in January, with a mean temperature of 42°; 33 in June, with a mean of 82°; 41 in July, with a mean of 82°; 38 in August, with a mean of 77°; and 16 in November, with a mean of 58°.

Such results show that temperature alone is not very closely connected with mortality, and that some other cause must be operative. The kinds of disease proving fatal also confirms this idea. Of cholera infantum there were only two deaths, one in June, and one in August.

The highest number of deaths by consumption were in August and September, being seven each month. The highest number of deaths by diarrhœa, was in July; the next highest being in May. October was most fatal to dysenteric cases. It is difficult to trace the effects of temperature in these statistics, and it becomes necessary to assign some other or allied agency.

On the whole this report is a very creditable one. If it is barren in statistical returns, it is because such were not within reach of the committee, and they seem to have carefully collected all important facts attainable, and to have given them their due share of consideration. H.

